# Stenting of the pancreatic duct in the early phase of acute pancreatitis: a retrospective study

**DOI:** 10.1186/s12876-022-02494-5

**Published:** 2022-09-10

**Authors:** Weijie Yao, Genwang Wang, Qi Wang, Feng Wang, Zuoquan Wang, Zuozheng Wang

**Affiliations:** 1 Department of Hepatobiliary Surgery, General Hospital of Ningxia Medical University, 804 Shengli South Street, Xingqing District, Yinchuan, 710004 China; 2 Department of General Surgery, The Third Affiliated Hospital of Xi’an Medical University, 277 Youyi West Road, Beilin District, Xi’an, 710068 China

**Keywords:** Acute pancreatitis, Endoscopic retrograde cholangiopancreatography, Pancreatic duct stenting, Pancreatic duct hypertension

## Abstract

**Background:**

The effectiveness of pancreatic duct (PD) stenting in the early stages of acute pancreatitis (AP) remains controversial. This study aimed to investigate the efficacy and safety of PD stenting in the early stages of AP.

**Methods:**

This is a retrospective cohort study. The clinical data of 131 patients with AP from 2018 to 2019 were analysed and divided into two groups: the study group (n = 46, PD stenting) and the control group (n = 85, standard treatment).

**Results:**

There was a statistically significant reduction in pain relief, oral refeeding, hospitalization, and intensive care unit (ICU) stay in the study group compared with that of the control group (*P* < 0.05). There were no significant differences in the incidence of complications between the two groups. Further multivariate analysis of risk factors for new-onset organ failure showed that the control group (odds ratio [OR] (95% confidence interval [CI]): 6.533 (1.104–70.181)) and a higher level of haematocrit (HCT) at admission (HCT > 46.1%, OR (95%CI): 8.728 (1.264–116.767)) were independent risk factors.

**Conclusions:**

In the early phase of AP, PD stenting has the potential to reduce pain relief time, oral refeeding time, ICU stay time, and overall hospital stay time. This finding highlights a new route for the treatment of AP.

**Supplementary Information:**

The online version contains supplementary material available at 10.1186/s12876-022-02494-5.

## Background

Acute pancreatitis (AP) is one of the most common digestive diseases and poses a serious threat to human health. AP is frequently accompanied by intense pain and organ malfunctions, resulting in high medical costs and healthcare issues [1]. AP has a high rate of morbidity and mortality worldwide [2–4]. The annual incidence of AP ranges from 13 to 45 per 100,000 worldwide, and in China, the incidence of AP has increased from 0.19 to 0.71% in the past 20 years. The overall mortality of AP varies from 1 to 5%, and can be as high as 20–35% in patients with severe AP [5–8].

AP can cause local and systemic inflammatory reactions and can present with various clinical outcomes depending on the degree of illness [2–4, 7]. In the early phase, it is characterised by acute sterile inflammation of the pancreas, with severe cases presenting with systemic disorders. In turn, patients present with systemic inflammatory response syndrome (SIRS), and in more severe cases, even multiple organ failure (MOF), which is the main cause of death [2]. According to current guidelines [9–11], vigorous intravenous hydration, pain control, correction of electrolyte and metabolic imbalances, and nutritional assistance are all part of the early phase of AP care.

Theoretically, pancreatic duct (PD) stent placement can reduce ongoing pancreatic injury by providing adequate drainage of pancreatic fluid. Animal studies revealed that both a blocked common bile duct and a blocked PD, or a blocked PD alone, could cause necrotising pancreatitis [12]. Continuous PD drainage can reduce the extravasation of fluid rich in pancreatic juice. One report pointed out that the rate of amylase elevation in the early peripancreatic puncture fluid in patients with moderately severe and severe AP was as high as 90% [13]. However, whether the placement of a PD stent can prevent or reduce subsequent complications is a topic that is hotly debated. A few studies have reported that the placement of PD stents is effective in relieving PD obstruction, reducing mortality, and decreasing the incidence of serious complications in the early phase of AP [14, 15]. Conversely, a prospective randomised study showed that PD stenting in acute necrotising pancreatitis was associated with a higher incidence of pancreatic necrosis infection [16]. Thus, the effectiveness of early PD stenting in patients with AP remains controversial. Even though the British Society of Gastroenterology guidelines for PC pain therapy recommend that PD stenting should be frequently used to treat chronic pancreatitis, it is rarely used to treat malignant strictures [17].

Yao et al. [18] demonstrated that in patients with AP, early endoscopic pancreatic stenting within 48 h after admission effectively reduces the fasting time and length of hospital stay. The rates of complications related to the procedure, in-hospital mortality, and local complication rates were 0.3%, 0.3%, and 7.6%, respectively [18].

Several investigations using PD stenting in the early phase of AP have recently been published with promising results [18, 19]. During the endoscopic operation, thick mucus or solid white materials were observed, blocking the PD in the early phase of AP. Early PD stenting also decreased the length of hospital stay and improved prognosis by promptly alleviating clinical symptoms. As a result, in this study, we retrospectively analysed a series of cases treated with PD stenting in the early phase of AP to confirm the feasibility and safety of PD stenting for patients with AP and to compare the application prospects of PD stenting in the management of patients with AP with a control cohort.

## Methods

### Patients

Patients with AP from 1 January 2018 to 31 December 2019 who underwent treatment at the General Hospital of Ningxia Medical University were included in this retrospective study. The inclusion criteria were as follows: 1) definitively diagnosed AP; 2) aged between 18 and 70 years; 3) onset time of no more than 72 h; 4) Acute Physiology and Chronic Health Evaluation (APACHE) II score ≥ 8 or C-reactive protein level (CRP) ≥ 150 mg/L; and 4) endoscopic retrograde cholangiopancreatography (ERCP) performed at the request of the patient. Exclusion criteria were: 1) pregnancy or lactation; 2) acute attack of chronic pancreatitis; 3) severe heart, lung, liver, kidney, and mental diseases that may impair AP treatment effect; 4) inability to cooperate during ERCP, NYHA cardiac function > III, acute myocardial infarction (AMI), creatinine (Cr) clearance rate of chronic kidney disease < 40 mL/min, chronic obstructive pulmonary disease patients who required long-term oxygen therapy, and patients with chronic liver disease with Child-Pugh liver function grade C, coagulation dysfunction or long-term oral anticoagulants, or the agonal phase; 5) patients with immune disorders or immunosuppression; 6) patients who cannot tolerate PD stenting for various reasons, such as hypersensitivity to contrast media, severe fundal oesophageal varices, upper digestive tract obstruction, ulcer, or bleeding; 7) malignant tumours of the biliary and pancreatic systems affecting the therapeutic effect; 8) refusal by the patient or family member; and 9) patients with other diseases requiring simultaneous surgical treatment. Ultimately, 131 patients (88 males and 43 females) who met the above criteria were classified into two groups according to whether they underwent PD stenting or not. The criteria for diagnosing and classifying AP were based on the 2012 Atlanta criteria [20].

### Treatment and procedure

All patients received standardised treatment on admission, in accordance with the guidelines developed by the American Gastroenterological Association (AGA) Institute (9). On this basis, patients in the PD stenting group (study group) underwent PD stenting within 48 h of admission.

The endoscopic procedure was similar to that previously described [18]. In short, after patients fasted for more than 12 h, 15 min before the procedure, 80 mg of intramuscular phloroglucinol (to inhibit the movement of smooth muscle that may affect intestinal peristalsis) and 2% lidocaine glue were administered. In the left supine position, the patient was given an intravenous injection of propofol 1.5–2.0 mg/kg and dezocine 0.2 μg/kg. The procedure was initiated after the patient’s eyelash reflex disappeared. Anaesthesia was maintained using continuous intravenous propofol (4–10 mg/kg/h) and dexmedetomidine (0.4 μg/kg/h).

Wire-guided cannulation over a pancreatic stent (WGC-PS) was used for biliary cannulation. An Olympus TJF-260 V, JF-260 V electronic duodenoscope, an Olympus disposable high-frequency papillotomy (KD-V411M-720), a COOK® band guidewire (ACRO-35-450), and a COOK® PD stent (according to the degree of oedema of the PD and the depth of cannulation; outside diameter 5–7 Fr and length 4–12 cm) were used in endoscopic surgery. The guidewire was gently introduced from the ampulla and validated under fluoroscopy in the direction of the PD once the duodenoscope reached the duodenum. To confirm the intrapancreatic position, a sphincterotome was introduced into the PD for aspiration until pancreatic fluid was detected. A pancreatic stent was implanted in the primary PD. Endoscopic retrograde cholangiography (ERC) and biliary drainage were performed simultaneously if the patient was suspected of having a biliary system illness. If necessary, a sphincterotomy was performed. To prevent post-ERCP pancreatitis, routine rectal administration of 100 mg diclofenac immediately after ERCP was performed according to the guidelines.

Endoscopists with more than 10 years of expertise, who have conducted more than 500 ERCP procedures every year and 500 PD cannulations in total, performed all the surgeries.

### Outcome indicators

#### Primary endpoints

Complications such as ERCP-related adverse events, new-onset organ failures, new systemic complications, late local complications, overall complications (local and systemic), and disease outcome within two months were considered to be the most important endpoints.

#### Secondary endpoints

Indicators such as abdominal pain relief time, oral refeeding time, length of hospitalisation, intensive care unit (ICU) stay, and several laboratory indicators were considered as secondary endpoints. Serum amylase (AMS), serum lipase (LPS), blood urea nitrogen (BUN), Cr, lactate dehydrogenase (LDH), white blood cells (WBC), and haematocrit (HCT) at 0, 48, and 72 h after admission were measured. The diagnostic criteria for AP include characteristic abdominal pain, elevated AMS or LPS, and radiographic evidence of pancreatitis. Serum concentrations of AMS and LPS increase within a few hours of pancreatic injury. In alcohol-induced pancreatitis, serum lipopolysaccharide (LPS) is the test of choice. However, neither enzyme can be used to monitor or predict the severity of pancreatitis attacks in adults [21].

### Data cleaning and processing

The character data of the original data were encoded, and the missing value statistics of the original data were obtained. For the missing objective indicators, the median of the same indicator was used, and the variable assignments performed during the analysis are described in the appendix.

### Ethical aspects

The Medical Research Ethics Review Committee of the General Hospital of Ningxia Medical University (No. 2019-467) approved this study, which followed the ethical criteria outlined in the Declaration of Helsinki (revised in 2013), and all patients provided informed consent. This was a single-centre, retrospective, case-control study (ChiCTR1900025833).

### Statistical analysis

The t-test or corrected t-test was used to analyse the mean ± standard deviation (mean ± SD) of continuous data, whereas the U test was used to analyse continuous data that did not follow a normal distribution in the form of the median (lower quartile–upper quartile) (median (IQR). The chi-square test or Fisher’s exact probability method was employed to assess the classified data (%) for comparison between groups.

The incidence of new-onset organ failure, which is extremely heterogeneous, was optimised using the first penalty maximum likelihood estimation, which can reduce the bias of the maximum likelihood estimation for small samples or sparse data by adding a penalty term. With new-onset organ failure as the dependent variable and the basic demographic characteristics, baseline situation, and intervention mode as independent variables, variables with *P* < 0.1 in univariate analysis were included in multivariate analysis, and variables were screened using the two-way stepwise regression method of the Akaike information criterion (AIC).

Multivariate analysis of indicators was performed based on the variable assignment table (Additional file 1: Table S1). R 3.6.3 or SAS 9.4 was used to assess all of the above tests, with *P* < 0.05 being regarded as statistically significant in the two-tailed test.

## Results

**Fig. 1:**
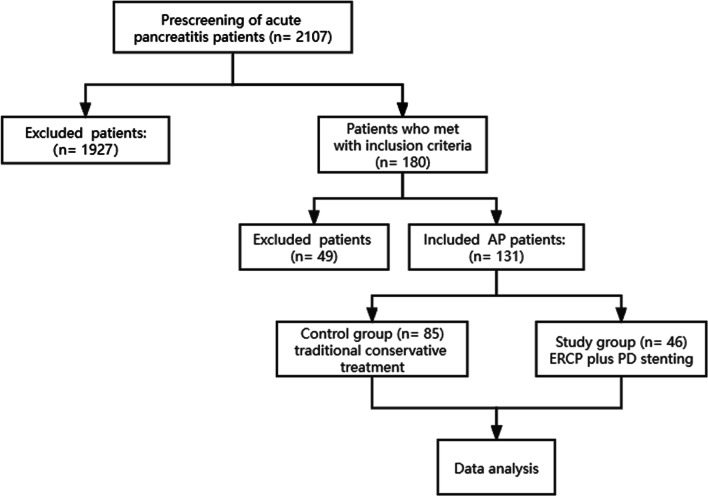
The flowchart of patient inclusion and pathways in this study

A total of 131 patients who met the inclusion criteria were enrolled in this study, including 46 in the PD stenting group (study group: ERCP-PD) and 85 in the control group (11 patients who underwent ERCP, Fig. 1). All patients in the study group successfully completed PD stenting.

### The comparison of baseline indicators

Of the 131 patients, 43 were women and 88 were men. The patients were aged 19–70 years, with a median age of 49.0 (IQR: 37.0, 64.0). APACHE II showed a score from 0 to 36, with a median score of 8.0 (IQR: 5.0, 10.0); C-reactive protein levels ranged from 143 to 473 mg/L, with a median level of 221.50 mg/L (IQR: 186.75, 277.00). The incidence of local complications at admission was significantly different between the two groups (*P* = 0.027). Importantly, the proportion of patients with pancreatic necrosis was higher in the study group than in the control group. The other baseline indicators were comparable between the two groups (Table 1).

**Table 1:** Sociodemographic and baseline admission characteristics of patients with AP

Indicators (Median (IQR))	Study group (N = 46)	Control group (N = 85)	Total (N = 131)	*P* value
Gender, n (%)				0.726^1^
Male	30 (65.2)	58 (68.2)	88 (67.2)	
Female	16 (34.8)	27 (31.8)	43 (32.8)	
Age (Y)	58.0 (41.0, 64.0)	47.0 (35.0, 65.0)	49.0 (37.0, 64.0)	0.301^2^
APACHE II Score	9.0 (6.0, 11.0)	8.0 (5.0, 10.0)	8.0 (5.0, 10.0)	0.266^2^
CRP	259.5 (190.25, 286.50)	213.0 (183.64, 269.00)	221.5 (186.75, 277.00)	0.277^2^
Local complications (admission), n (%) (0-none; 1-peripancreatic fluid collection; 2-peripancreatic necrosis; 3-pancreatic necrosis)				0.027^1; *^
0	17 (37.0)	21 (24.7)	38 (29.0)	
1	10 (21.7)	38 (44.7)	48 (36.6)	
2	6 (13.0)	14 (16.5)	20 (15.3)	
3	13 (28.3)	12 (14.1)	25 (19.1)	
CT acute pancreatitis grade score	3.00 (1.25, 4.00)	3.00 (2.00, 4.00)	3.00 (2.00, 4.00)	0.698^2^
CT pancreatic necrosis degree score	0.00 (0.00, 2.00)	0.00 (0.00, 0.00)	0.00 (0.00, 0.00)	0.098^2^
Systemic complications (admission), n (%)				0.721^1^
	28 (60.9)	49 (57.6)	77 (58.8)	
Organ failure, n (%)				0.052^1^
	26 (56.5)	33 (38.8)	59 (45.0)	
Acute respiratory failure, n (%)				0.206^1^
	22 (47.8)	31 (36.5)	53 (40.5)	
Acute renal failure, n (%)				> 0.999^3^
	3 (6.5)	7 (8.2)	10 (7.6)	
Acute circulatory failure, n (%)				0.740^3^
	4 (8.7)	6 (7.1)	10 (7.6)	
Biliary causes, n (%)	28 (60.9)	29 (34.1)	57 (43.5)	0.003^1; **^
BUN (admission), mmol/L	6.0 (4.5, 7.5)	5.4 (3.9, 6.6)	5.6 (4.1, 7.1)	0.083^2^
Cr (admission), μmol/L	72.6 (53.8, 90.8)	66.1 (57.4, 85.0)	68.2 (55.9, 85.3)	0.939^2^
LDH (admission), U/L	850.0 (499.0, 1286.0)	702.0 (445.0, 1061.0)	706.0 (483.0, 1132.0)	0.161^2^
AMS (admission), U/L	1016.1 (522.7, 1541.0)	436.2 (189.3, 922.7)	623.1 (270.8, 1117.7)	< 0.001^2; ***^
LPS (admission), U/L	4645.0 (1976.0, 8007.0)	2000.0 (643.0, 7555.0)	3140.0 (1087.7, 7787.0)	0.063^2^
WBC (admission), × 10^9^/L	14.7 (11.2, 18.6)	15.6 (12.5, 19.2)	15.2 (11.7, 19.0)	0.345^2^
HCT (admission), %	46.7 (41.1, 51.1)	45.1 (40.4, 49.7)	46.1 (41.1, 50.6)	0.201^2^

^1^Chi-Square *p*-value; ^2^Mann-Whitney U *p*-value; ^3^Fisher Exact *p*-value

*P < 0.05, **P < 0.01, ***P < 0.001

### The comparison of primary endpoints

With the incidence of complications as the primary endpoint, no procedure-related adverse events occurred. There was only one (2.2%) new-onset organ failure in the study group and 11 (12.9%) new-onset organ failures in the control group, a difference of 10.7% in the incidence rate between the two groups. However, there was no significant difference in the incidence of other complications between the two groups (*P* = 0.085, Table 2). There was no significant difference between the two groups in terms of the incidence of other complications.

**Table 2:** Comparison of incidence rate of complications

Indicator	Study group (N = 46)	Control group (N = 85)	Total (N = 131)	*P* value
New-onset organ failure, n (%)				0.085^1^
	1 (2.2)	11 (12.9)	12 (9.2)	
New-onset systemic complications, n (%)				0.244^2^
	8 (17.4)	24 (28.2)	32 (24.4)	
Late local complications, n (%) (0- none; 1- pseudocyst; 2- wall-off necrosis; 3- infectious necrosis)				0.959^2^
0	33 (75.0)	62 (73.8)	95 (74.2)	
2	4 (9.1)	9 (10.7)	13 (10.2)	
3	7 (15.9)	13 (15.5)	20 (15.6)	
Critical complex end point, n (%) (0 = cure; 1 = asymptomatic/symptomatic complications/death)				0.701^2^
0	37 (82.2)	66 (77.6)	103 (79.2)	
1	8 (17.8)	19 (22.4)	27 (20.8)	

^1^Yates’ continuity correction; ^2^Chi-Square *p*-value

### The comparison of second endpoints

The comparison of pain relief time, oral refeeding time, length of hospital stay, and ICU stay.

As shown in Table 3, the median pain relief time in the study group was two days (IQR: 1, 3), which was two days shorter than that in the control group (4 (IQR: 2, 7), *P* < 0.001), and the median oral refeeding time was three days shorter in the study group than in the control group (4 (IQR: 2, 6) versus 7 (IQR: 5, 10), *P* < 0.001). Additionally, the median length of stay in the study group was four days shorter than that in the control group (six days (IQR: 5, 11) versus 10 days (IQR: 8, 13), *P* = 0.005). Overall, there were 13 patients admitted to the ICU in both groups, and there was no difference in the ICU occupancy rate between the two groups. The median length of ICU stay was five days shorter in the study group than in the control group (5 (IQR: 2, 7) vs. 10 (IQR: 7, 16), *P* = 0.039).

**Table 3:** The comparison of pain relief time, oral refeeding time, length of hospital stay and ICU stay

Indicators (days)^1^	Study group (N = 46)	Control group (N = 85)	Total (N = 131)	*P* value
Time of pain relief	2 (1,3)	4 (2,7)	3 (2,6)	< 0.001^***^
Time of oral refeeding	4 (2,6)	7 (5,10)	6 (4,9)	< 0.001^***^
Length of hospital stay	6 (5,11)	10 (8,13)	10 (6,13)	0.005^**^
ICU care, n (%)	5 (10.9)	8 (9.4)	13 (9.9)	> 0.999^2^
Length of ICU stay	5 (2,7)	10 (7,16)	7 (5,10)	0.039^*^

^1^The indicators of pain relief time, time to resume oral feeding, length of hospital stay and length of ICU stay are all analyzed by the corresponding median value (IQR); ^2^Fisher exact *p*-value. (*P < 0.05, **P < 0.01, ***P < 0.001)

### The comparison of laboratory indicators

On comparing the difference values of laboratory indicators from the baseline at 48 or 72 h between the two groups, the results showed that the changes in AMS between the two groups were statistically significant, with a more pronounced decrease in AMS in the study group than in the control group: 1) the median differences between the two groups at 48 h and admission baseline were − 697.10 (IQR: − 1209.53, − 255.60) and − 303.00 (IQR: − 792.90, − 76.90), *P* = 0.004, respectively, and 2) the median difference from the baseline at 72 h between the two groups was − 884.40 (IQR: − 1359.38, − 385.15) and − 398.40 (IQR: − 820.40, − 116.10), *P* = 0.003, respectively. However, there were no significant differences in the other biochemical indices between the two groups (Table 4).

**Table 4:** The comparison of laboratory indicators

Indicators	48 h after admission versus admission			72 h after admission versus admission		
	Study group	Control group	*P* value	Study group	Control group	*P* value
BUN	− 0.52 (− 1.54, 1.55)	− 0.70 (− 1.80, 0.58)	0.351	− 1.15 (− 1.96, 1.08)	− 0.51 (− 1.99, 1.02)	0.925
Cr	− 3.80 (− 15.53, 6.28)	− 4.00 (− 15.35, 4.35)	0.883	− 7.85 (− 24.03, − 0.75)	− 7.00 (− 22.60, − 0.10)	0.693
LDH	− 76.00 (− 395.12, 320.62)	− 98.00 (− 378.00, 128.00)	0.442	17.50 (− 377.00, 336.00)	− 114.00 (− 364.00, 185.00)	0.231
AMS	− 697.10 (− 1209.53, − 255.60)	− 303.00 (− 792.90, − 76.90)	0.004^**^	− 884.40 (− 1359.38, − 385.15)	− 398.40 (− 820.40, − 116.10)	0.003^**^
LPS	− 2457.00 (− 5096.00, − 1340.50)	− 2457.00 (− 3067.00, − 933.00)	0.690	− 2672.00 (− 5079.25, − 1775.50)	− 2672.00 (− 3077.00, − 1096.00)	0.282
WBC	− 3.38 (− 6.26, 0.25)	− 4.54 (− 8.00, − 1.05)	0.160	− 3.72 (− 8.37, − 0.91)	− 6.03 (− 9.12, − 1.94)	0.591
HCT	− 4.85 (− 9.18, − 2.30)	− 5.70 (− 10.10, − 2.10)	0.754	− 7.85 (− 12.45, − 4.65)	− 6.50 (− 10.25, − 3.00)	0.238

All results of the indicators were analyzed with median (IQR)

**P < 0.01

### Analysis of independent risk factors for new-onset organ failure

A univariate analysis was performed to compare the differences between the single-factor groups, and the indicators (*P* < 0.1) were selected as independent variables and included in the univariate analysis. The results indicated that indicators such as LPS (admission), HCT (admission), biliary pancreatitis, local complications at admission, and venous thrombosis may be independent risk factors.

Furthermore, a multivariate analysis of risk factors for new-onset organ failure was performed. Ultimately, three variables were retained using a two-way stepwise regression model based on the principle of AIC, including grouping, HCT (grouped by the median of 46.1), and local complications at the time of admission. Subsequently, the Hosmer–Lemeshow goodness-of-fit test was used to evaluate the model fitting, and the results showed *χ*^*2*^ = 3.3227, *P* = 0.9125, indicating good model fitting. In addition, the likelihood ratio test (*χ*^*2*^ = 18.167, *P* = 0.003) and Wald’s test (*χ*^*2*^ = 11.995, *P* = 0.035) both showed significance, indicating that the goodness of fit of the model was verified when at least one of these included independent variables had a coefficient that was not 0 (Additional file 1: Table S1).

In summary, when other covariables were controlled for, grouping indicators and HCT at admission were independent predictors of new-onset organ failure. As shown in Table 5, the risk of new-onset organ failure was 6.533 times higher in the control group than in the study group (95% confidence interval [CI]: 1.104–70.181, *P* = 0.038) and 8.728 times higher in patients with HCT above the median (46.1) than in those below the median (95% CI: 1.264–116.767, *P* = 0.027).

**Table 5:** Multivariate analysis of risk factors for new-onset organ failure

Indicators	Reference groups	Coefficient	SE	χ^2^	*P*	OR (95%CI) ^#^
Grouping	Study group	1.877	0.987	1.408	0.038^*^	6.533 (1.104 ~ 70.181)
HCT (admission) > 46.1%	≦46.1%	2.167	1.052	2.356	0.027^*^	8.728 (1.264 ~ 116.767)
Local complication (admission) 1	0	− 2.791	1.782	0.804	0.089	0.061 (0.0004 ~ 1.535)
Local complication (admission) 2	0	0.680	1.196	0.001	0.565	1.973 (0.182 ~ 26.083)
Local complication (admission) 3	0	− 0.094	1.276	0.105	0.941	0.910 (0.067 ~ 13.618)

^#^Means profile likelihood confidence interval instead of the Wald confidence interval which commonly used in statistical software. When separation occurs, the distribution of parameters is no longer normal distribution, so the Wald method is no longer applicable, and contour likelihood confidence interval is recommended

*P < 0.05

## Discussion

During the exploration of AP, the leading view of its pathogenesis has been controversial. In 1901, Opie put forward the famous viewpoint, ‘common channel theory’, stating that the gallstone obstructed the common channel between the common bile duct and the PD, leading to bile reflux and activation of pancreatic enzymes, leading to pancreatitis [22]. Although the pathogenesis of AP has not been clarified clearly in recent years, biliary tract obstruction has been proven as one of the most important leading causes in the pathogenesis of AP [23]. However, studies have shown that similar necrotising pancreatitis can be induced by obstruction of the common bile duct and PD, as well as by obstruction of PD alone in animal models [12]. Therefore, the view that PD hypertension induced by PD obstruction and acinar hyperstimulation plays an important role in the initial stage of AP is more convincing [23–25]. Most studies agree that PD blockage and hypertension are important factors in the development and progression of AP [15, 26]. However, there has been little research on treating AP using pancreatic procedures to relieve PD blockage in the early stages [27].

Notably, normal or emergency ERCP in patients is not recommended by the latest AGA guidelines [9]. Although ERCP can unclog the PD and effectively drain the pancreatic fluid, it has no clear effect on clinical outcomes, such as mortality, the incidence of complications including organ failure, systemic complications, or pancreatic necrosis. During ERCP, most patients are under local anaesthesia. When the guidewire enters the PD and is accompanied by the flow of pancreatic fluid, it can experience a significant reduction in pain, suggesting pain caused by PD blockage [28].

In patients with a high surgical risk, ERCP for PD insertion is also worth trying if it can improve clinical outcomes. To avoid the risk of iatrogenic secondary infection caused by debridement or drainage, conservative schemes were recommended for patients in the early phase of AP [9, 10]. However, the early phase of AP was always accompanied by pain and discomfort. Importantly, pain is an independent risk factor that can determine whether patients can affect the overall recovery rate. The PD stenting regimen may play a vital role in reducing the secondary infection caused by the procedure and relieving pancreatic pain, thus accelerating the patient's recovery. Several retrospective and prospective studies have been performed in patients undergoing PD stenting to verify the efficacy and safety of the treatment of acute biliary pancreatitis (ABP) [14, 15] and post-ERCP pancreatitis [29]. The results showed that PD stenting was effective in reducing the risk of adverse events, death, or local complications, and improved the prognosis, which further indicated a novel safe and effective option for AP.

Our team has used PD stenting to treat AP since 2011. During the treatment procedure, patients with AP were found to have flocculent or solid particles that blocked pancreatic fluid drainage [18, 19]. In patients with AP, the obstructive state may be alleviated by PD stenting, which has good therapeutic results. Through case–control research, we evaluated whether PD stenting could improve the problems of patients with AP in this study. The findings revealed that there was only one case of new-onset organ failure (2.2%) in the study group and 11 instances (12.9%) in the control group, with a 10.7% difference in new-onset organ failure incidence between the two groups (*P* = 0.085). The main causes of death in the early phase of severe AP are systemic inflammation and organ failure. In patients with persistent multiple organ failure, the mortality rate will be > 30% [30]. Although the difference between the two groups was not statistically significant at *α* = 0.05, it is still intriguing. Thus, the risk factors were evaluated by multifactorial analysis, and the results indicated that the incidence of new organ failure was significantly lower in the study group than in the control group (odds ratio [OR] (95% CI): 6.533 (1.104–70.181)), controlling for other covariates. Meanwhile, the incidence of new-onset organ failure was much higher in patients with higher HCT levels than in those with lower levels. Since HCT has been used as a key indicator to assess the severity of AP and predict the prognosis of AP [31, 32], this outcome was consistent with clinical practice and showed that the results of the multifactor analysis were valid and credible.

There were no significant differences in other complications between the two groups. Although the proportion of patients with necrotising pancreatitis at admission was higher in the study group than in the control group (28.3% vs. 14.1%), the incidence of late local complications was similar in both groups (e.g., infected necrosis, 15.9% vs. 15.5%). Additionally, there were statistically significant differences between the study and control groups in terms of pain relief time, oral refeeding time, hospitalisation stay, and ICU stay. The variations in blood AMS at 48 or 72 h were statistically significant according to a comparison of clinical laboratory markers between the groups and differences in admission baseline, and the decrease in blood AMS was more pronounced in the study group than in the control group compared with the admission level. These outcomes indicated that patients in the PD stenting group had a faster recovery rate. Simultaneously, PD stenting in the early phase of AP did not cause procedure-related adverse events or an increased incidence of complications. Therefore, PD stenting in the early phase of AP may be safe and feasible. However, endoscopic intervention in critically ill patients with severe pancreatitis and papilledema may be technically challenging and may even worsen clinical outcome [12]. Therefore, only centres and physicians with extensive experience in PD cannulation should perform PD stenting procedures. It is important to be aware that, clinically, 80–90% of patients with AP have a minor clinical course. Patients with mild AP tolerate oral all-solid diets well and have shorter hospital stays than patients who do not have recurrent stomach pain [33, 34].

The present study was limited by the small number of cases and single-centre data, which may affect evaluation to some extent, and statistical methods such as multivariate analysis can only reduce the bias caused by confounding factors. Therefore, the efficacy evaluation of PD stenting in patients in the early phase of AP still needs to be verified further by a large-sample multicentre study. As PD stenting for AP is an ongoing exploration, we need to set more inclusion and exclusion criteria in the future to eliminate the heterogeneity between the two groups.

## Conclusions

The use of early PD stent implantation in the treatment of patients with AP may be advantageous for lowering the incidence of newly developed organ failure. To ensure safety during the early treatment of AP, the potential of HCT, which can alter the incidence of emergent organ failure, must not be overlooked. This could help in the diagnosis, treatment, and prognosis of early PD stenting. Additionally, the regimen can reduce pain relief time, oral refeeding time, ICU time, and total hospital stay time. Hence, we believe that PD stenting may be safe and effective in the early stages of AP treatment, but large prospective randomised clinical trials should be conducted to support this result.

## Supplementary Information

**Additional file 1**. Variable assignment for multivariate analysis.

## Data Availability

All data generated during this study are included in this published article.

## Abbreviations

PD: Pancreatic duct
AP: Acute pancreatitis
HCT: Hematocrit
SIRS: Systemic inflammatory response syndrome
MOF: Multiple organ failure
AGA: American Gastroenterological Association
ERC: Endoscopic retrograde cholangiography
