# Supplementary material for: Stenting of the pancreatic duct in the early phase of acute pancreatitis: a retrospective study
Source: BMC Gastroenterol. 2022 Sep 10;22:414. doi: 10.1186/s12876-022-02494-5 (PMC9463836; doi:10.1186/s12876-022-02494-5)
Supplement: Supplementary file 1 — Additional file 1. Variable assignment for multivariate analysis. [file 12876_2022_2494_MOESM1_ESM.docx]

# Additional file 1: table S1

## Table of variable assignments

| Variables | Assignment * |
| --- | --- |
| Pain relief time (dependent variable) | NRS pain score≦3 = 0; >3 = 1 |
| Hospital days (dependent variable) | ≦10 = 0; >10 = 1 |
| Baseline indicators (independent variable) | |
| Sex | Male = 1; female = 2 |
| Age (Y) | ≦49 = 0; >49 = 1 |
| Grouping | PD stenting group = 1; control group = 2 |
| CRP | ≦221.5 = 0; >221.5 = 1 |
| Systemic complications (admission) | Yes = 1; No = 0 |
| SIRS | Yes = 1; No = 0 |
| Sepsis | Yes = 1; No = 0 |
| Venous thrombosis | Yes = 1; No = 0 |
| Acute liver injury | Yes = 1; No = 0 |
| Abdominal compartment syndrome | Yes = 1; No = 0 |
| Organ failure | Yes = 1; No = 0 |
| Acute respiratory failure | Yes = 1; No = 0 |
| Acute renal failure | Yes = 1; No = 0 |
| Acute circulatory failure | Yes = 1; No = 0 |
| Biliary causes | Yes = 1; No = 0 |
| Blood Urea Nitrogen (admission) | ≦5.6 = 0; >5.6 = 1 |
| Creatinine (admission) | ≦68.2 = 0; >68.2 = 1 |
| Lactate dehydrogenase (admission) | ≦706 = 0; >706 = 1 |
| Amylase (admission) | ≦623.1 = 0; >623.1 = 1 |
| Lipase (admission) | ≦3140 = 0; >3140 = 1 |
| Leukocyte (admission) | ≦15.2 = 0; >15.2 = 1 |
| Hematocrit (admission) | ≦46.1 = 0; >46.1 = 1 |

*: Several continuous variables take the median as the cutoff value and are converted into dichotomous variables.
